# Biomarkers (mRNAs and non-coding RNAs) for the diagnosis and prognosis of rheumatoid arthritis

**DOI:** 10.3389/fimmu.2023.1087925

**Published:** 2023-02-01

**Authors:** Yong Jiang, Shuxin Zhong, Shenghua He, Juanling Weng, Lijin Liu, Yufeng Ye, Hanwei Chen

**Affiliations:** ^1^ Department of Radiology, Guangzhou Panyu Central Hospital, Guangzhou, China; ^2^ Graduate School, Guangzhou University of Chinese Medicine, Guangzhou, China; ^3^ Department of Medical Biochemistry and Molecular Biology, School of Medicine, Jinan University, Guangzhou, China; ^4^ The Fourth Clinical Medical College of Guangzhou University of Chinese Medicine, Shenzhen, China; ^5^ Department of Radiology, GuangzhouPanyu Health Management Center (Panyu Rehabilitation Hospital), Guangzhou, China

**Keywords:** rheumatoid arthritis, biomarkers, mRNA, non-coding RNA, prognosis, diagnosis

## Abstract

In recent years, diagnostic and therapeutic approaches for rheumatoid arthritis (RA) have continued to improve. However, in the advanced stages of the disease, patients are unable to achieve long-term clinical remission and often suffer from systemic multi-organ damage and severe complications. Patients with RA usually have no overt clinical manifestations in the early stages, and by the time a definitive diagnosis is made, the disease is already at an advanced stage. RA is diagnosed clinically and with laboratory tests, including the blood markers C-reactive protein (CRP) and erythrocyte sedimentation rate (ESR) and the autoantibodies rheumatoid factor (RF) and anticitrullinated protein antibodies (ACPA). However, the presence of RF and ACPA autoantibodies is associated with aggravated disease, joint damage, and increased mortality, and these autoantibodies have low specificity and sensitivity. The etiology of RA is unknown, with the pathogenesis involving multiple factors and clinical heterogeneity. The early diagnosis, subtype classification, and prognosis of RA remain challenging, and studies to develop minimally invasive or non-invasive biomarkers in the form of biofluid biopsies are becoming more common. Non-coding RNA (ncRNA) molecules are composed of long non-coding RNAs, small nucleolar RNAs, microRNAs, and circular RNAs, which play an essential role in disease onset and progression and can be used in the early diagnosis and prognosis of RA. In this review of the diagnostic and prognostic approaches to RA disease, we provide an overview of the current knowledge on the subject, focusing on recent advances in mRNA–ncRNA as diagnostic and prognostic biomarkers from the biofluid to the tissue level.

## Introduction

Rheumatoid arthritis (RA) is the most common chronic systemic autoimmune disease. Its etiology is unknown. The current global prevalence of RA, increasing over time (1), is approximately 0.5% to 1%. Occurring primarily in women, RA is associated with considerable disability and mortality, presenting a serious public health problem (2). The prognosis of RA is closely associated with the disease stage at the time of diagnosis. The lack of treatment in the setting of a late RA diagnosis leads to serious systemic disease with systemic multi-tissue and multi-organ damage, with a consequent high disability, mortality, and negative socioeconomic consequences (3). On the other hand, the early diagnosis and treatment of RA can prevent or significantly delay disease progression in up to 90% of patients, thereby preventing irreversible joint damage and disability (4).

The ability to detect reliable RA biomarkers early would be a promising medical advantage, shifting the “window of opportunity” to the preclinical phases of RA (5). These markers can be utilized to identify the early stages or susceptibility to the disease and to monitor the effects of treatment during the course of the disease, thereby determining the prognosis of the patient. In addition, those who undergo early screening may benefit from active early treatment, and patients at high risk of developing RA could receive preventive interventions to reduce the risk of RA progression from an indiscriminate inflammatory arthritis to classifiable RA (6), minimize RA risk factors, and adjust treatment regimens based on frequent surveillance results.

## Molecular pathogenesis of RA

Although RA develops with genetic and epigenetic components, environmental factors also play an important role (7). Gene–environment interactions trigger autoimmune dysregulation (8), and sustained immune cell activation leads to a chronic inflammatory state. Progressive accumulation results in the loss of joint function and systemic multi-tissue organ damage. Studies have estimated the genetic risk of RA to be approximately 50%, and two types of RA can be classified according to the presence or absence of ACPA, with associated differences in risk factors, including ACPA+ patients showing a higher correlation with genetic factors (9).

Ubiquitous RA-specific autoantigens cannot be completely removed, and antigens modified by citrullination, acetylation, and carbamylation trigger antibody responses relevant to RA pathogenesis (10, 11). These autoantibodies form immune complexes that attract immune cells (12), which is believed to be the principal molecular mechanism contributing to RA pathogenesis. RA is a highly heterogeneous disease because of molecular variation in primary genetic factors and the various expression patterns of synovial tissue, as well as the heterogeneity of cells associated with RA pathogenesis [e.g., fibroblast-like synoviocytes (FLSs), macrophages, monocytes, and mast cells] (13). This heterogeneity in the molecular pathogenesis of RA is important in clinical practice because identifying these subtypes with different subtype-specific genetic markers can direct the “precision individualized diagnosis and treatment management” of RA patients. In addition, clinical monitoring of RA symptoms can improve patients’ physical and mental health (14).

## Current diagnostic and prognostic methods of RA

According to the ACR/EULAR (the American College of Rheumatology and the European League Against Rheumatism) 2010 RA classification criteria, the diagnosis of RA requires patients to have swelling in at least one joint on clinical examination. Confirmation is followed by a sensitive assessment of the involved joint (those with tenderness, with positive plain film/CT, or ultrasound or MRI are classified as active), combined with serological biomarkers (RF and ACPA) and acute phase reactants (ESR and CRP). Finally, a scoring system is applied (patients with a score ≥6 are classified as having RA). However, when the imaging shows RA erosion features, the scoring system may not be applied, and RA can be classified directly (1, 15). The original impetus for the RA classification criteria was to include patients in the early stages of the disease so they could benefit from early and active treatment. Before reaching the clinical symptom phase, the patient has gone through the preclinical “healthy life” phase, and the pathophysiological changes of RA have occurred throughout the body without treatment (16).

All of these diagnostic methods have certain limitations. Ultrasound is an operator skill- and experience-dependent technology in terms of measurement and quality evaluation (17). Plain film/CT examinations can be harmful with ionizing radiation and have limited soft tissue contrast (18). Although MRI is highly accurate for early RA detection, it is limited by the cost of routine use and the inability to image multiple sites with a single test (19). For laboratory tests, ESR and CRP are usually used to check the general inflammatory status of patients, and RF and ACPA are found in RA and healthy donors and patients with other diseases; notably, ACPA is harmful in some RA patients (20). These laboratory tests are used in clinical practice, but their sensitivity and specificity are moderate and have limited value for early diagnosis, subtype classification, and prognosis (21). Therefore, many studies used biofluids or tissues to establish innovative screening programs targeting abnormal proteins, mRNA expression, genetic variation, and epigenetic variation (e.g., DNA methylation, histone modification, ncRNA, bromodomain, and sirtuin) (22–26). Identifying molecular markers based on protein, DNA, or RNA to develop novel non-invasive or minimally invasive blood or tissue RA biomarker detection methods has become a worldwide research focus (22, 27–29).

Many genome-wide association studies (GWAS) have identified genetic factors and the molecular variation underlying them (30–33), with the most evident aspects including class II human leukocyte antigen (HLA) genes (e.g., *HLA-DRB1*), protein tyrosine phosphatase non-receptor 22 (*PTPN22*), peptidyl arginine deiminase type IV (*PADI4*) (34), chemokine receptor genes (e.g., *CCR6*), signal transducer and activator of transcription 4 protein (*STAT4*), cytotoxic T-lymphocyte antigen 4 (*CTLA4*), and the B-cell cell surface receptor gene (*CD40*) (35). These genetic factors predispose individuals to RA and may serve as a susceptibility criterion for early RA diagnosis (13). Similarly, in another GWAS comprising 262 ACPA-negative early RA patients, 33 single nucleotide polymorphisms (SNPs) were shown to be associated with joint destruction, with rs2833522 being related to the severity of bone destruction (36). In addition, the GWAS analysis of 457 RA patients’ response to methotrexate (MTX) therapy identified 10 novel risk loci associated with a poor response to MTX, of which thymidylate synthase (*TYMS*), dihydrofolate reductase (*DHFR*), folylpolyglutamate synthetase (*FPGS*), and enolase superfamily member 1 (*ENOSF1*) were validated genes (37). In a GWAS analysis of 2,706 RA patients, Ming Li et al. identified an SNP (rs6427528) at the 1q23 locus that was related to changes in the disease activity scores of patients undergoing etanercept [an anti-tumor necrosis factor-α (TNF-α) drug] treatment. This SNP could disrupt transcription factor binding site motifs in the 3′UTR of *CD84* (an immune-related gene), and the allele correlated with a better etanercept response was related to higher *CD84* gene expression levels in peripheral blood mononuclear cells (38). Moreover, other studies have shown that the rs7195994 variant at the fat mass and obesity-associated protein (FTO) gene locus was associated with an improved clinical response to infliximab (39) and that the protein tyrosine phosphatase receptor type C (PTPRC) rs10919563 SNP was relevant to having an excellent response to anti-TNF-α therapy in RA patients (40).

Sperm-associated antigen 16 (SPAG16) has a protective effect on the joints by influencing the regulation of matrix metalloproteinase-3 (MMP-3) in autoantibody-positive RA and is associated with a good prognosis in RA patients (23). Elevated serum 14-3-3η protein was associated with more serious joint erosion and worse treatment outcomes in RA patients. It could be used as a biomarker to assess the diagnosis, prognosis, and therapy response (41). In addition, serum soluble folate receptor β (sFRβ) levels could act as a biomarker of disease activation and the anti-TNF drug response (42). Studies have shown that the C-terminal telopeptide of collagen type I (CTX-I) and CTX-II in biofluids could be used as markers of bone resorption and cartilage degradation in RA, respectively, to predict the degree of joint damage and monitor the therapy response (43). A large study showed that serum calcineurin levels correlate with disease activity and severity in RA (44). A multicenter study identified soluble scavenger receptor-A (sSR-A) as a potential diagnostic biomarker and therapeutic target of RA and fibrinogen-like protein 1 (FGL1) as a specific biomarker that could help predict RA progression (45, 46). The four-biomarker panel [serum amyloid A-4 protein (SAA4), retinol-binding protein-4 (RBP4), vitamin D-binding protein (VDBP), and angiotensinogen (AGT)], autoantibodies against peptidoglycan recognition protein-2 (PGLYRP-2), and lipopolysaccharide-binding protein (LBP) could be promising serum biomarkers for early diagnosis and disease activity assessment in seronegative RA patients (47–49).

A study predicting the anti-TNF-α drug response of RA patients by machine learning using the Dialogue on Reverse Engineering Assessment and Methods (DREAM) to validate and evaluate patient data correctly categorized responses from 78% of patients and found that specific genetic markers were shared by distinct populations and identifying them could improve the prediction of anti-TNF-α therapy efficacy (50).

Furthermore, the following are some examples of currently used and well-studied biomarkers that play a crucial role in the diagnosis and prognosis of RA: acute phase (serum amyloid A, ferritin, and procalcitonin), antibody [antibodies against v-RAF murine sarcoma viral oncogene homolog B (BRAF), antibodies against peptidyl arginine deiminase 4 (PAD4), anti-mutated citrullinated vimentin antibodies, and anti-carbamylated and anti-acetylated protein antibodies], pathogenesis- and bone metabolism-related [interleukin-6 (IL-6)/interleukin-1β (IL-1β)/TNF-α, connective tissue growth factor (CTGF), leucine-rich alpha2 glycoprotein (LRG), Krebs von den Lungen-6 (KL-6), vascular cell adhesion protein 1 (VCAM1), vascular endothelial growth factor (VEGF)/EGF, MMP1/MMP3, C-X-C motif chemokine ligand 13 (CXCL13)/CXCL16/chitinase-3-like-1 protein (YKL-40), and soluble intercellular adhesion molecule-1 (sICAM1)] (20, 26, 51). Currently, the diagnostic test markers for RA also include ESR, CRP, RF, ACPA, serum DNA, cell-free nucleic acid, histone modification, and other circulating DNA methylation biomarkers (hypermethylated genes: *DUSP22*, *DR3*, *IL-10*; hypomethylated genes: *IL-6*, *STA3*, *STAT4*, *CXCL12*, *IFIH1*, *DUSP22*, *IRF5* (52), *mRNA*, and *ncRNA*) (53).

## Candidate RNAs as biomarkers for RA

The complete analysis of the whole human genome has shown that nearly 70%–90% of the genome has been transcribed into RNA (54). Only 1.1% of the genome comprises coding sequences, and approximately 24% has been transcribed into pre-mRNAs with introns. Finally, ncRNAs are transcripts explaining the role of the remaining 75% of the genome (55, 56). The biological importance of ncRNAs has been demonstrated by their discovery in almost all joint tissues and biofluids of different species. Furthermore, ncRNAs could act as master regulators of gene expression in a series of biological processes such as epigenetic, transcriptional, splicing, and translation. The specific expression profiles of ncRNAs in various disease states support their roles as mediators of pathogenic mechanisms, potential therapeutic targets, and promising candidate biomarkers (57) and their extensive involvement in the development and progression of many diseases, including RA (58).

The ncRNAs are divided into two major categories: housekeeping ncRNAs comprise transfer RNA (tRNA), ribosomal RNA (rRNA), small nucleolar RNA (snoRNA), and small nuclear RNA (snRNA), and regulatory ncRNAs, which are involved in regulating transcription and RNA processing and translation, comprise long non-coding RNA (lncRNA), circular RNA (circRNA), microRNA (miRNA), small interfering RNA (siRNA), and Piwi-interacting RNA (piRNA) (59–61).

### mRNAs as biomarkers for RA

mRNAs are transcribed from DNA, carry genetic information, and act as templates in protein synthesis (62). In a study including 130 RA patients, semaphorin 3A (Sema3A) mRNA expression was 1.8-fold higher in peripheral blood mononuclear cells (PBMCs) of RA patients than in healthy controls (HCs). It was correlated with RF, immunoglobulin M (IgM), ESR, platelet counts, lumbar spine bone mineral density (BMD), and the Sharp score. The optimal diagnostic cutoff value of 10.881 ng/ml for Sema3A was based on the receiver operating characteristic (ROC) curve (63). In addition, ribophorin-II (RPN2) mRNA expression was significantly upregulated in the PBMCs of RA patients in a case–control study sample. The *RPN2* gene affects the growth and activation of T lymphocytes and is involved in the pathogenesis of RA; it could serve as a novel biomarker for RA diagnosis (64). IL-37 mRNA levels in the plasma of RA patients in the training cohort were measured by reverse transcription quantitative PCR (RT-qPCR) and found to be significantly increased compared with HCs. The levels were also correlated with 28-Joint Disease Activity Score (DAS28)-ESR and CRP, which have good diagnostic ability to predict RA [area under the curve (AUC) = 0.97]. Furthermore, in a validation cohort of 598 patients comprising 230 RA patients, this finding suggested a higher specificity of IL-37 in identifying RA compared with patients with OA (AUC = 0.87), systemic lupus erythematosus (SLE) (0.86), gout (0.91), ankylosing spondylitis (AS) (0.92), and primary Sjögren’s syndrome (pSS) (0.87) (65). A significant inverse association between the suppressor of cytokine signaling 1 (SOCS1) mRNA expression levels in the PBMCs of RA patients and disease activity was seen in four independent patient cohort studies comprising 281 RA patients, a finding that can guide prognostic stratification and treatment decisions (66).

A study including 65 RA patients showed that hexokinase-2 (HK2) mRNA levels in PBMCs were positively associated with Clinical Disease Activity Index (CDAI), DAS28-ESR, and Simplified Disease Activity Index (SDAI) scores, independently correlated with increased disease activity risk, and may be involved in the molecular mechanisms of RA, and that HK2 could be a prospective candidate marker for RA diagnosis (RA vs. HCs, AUC = 0.808; RA vs. OA, AUC = 0.655) (67). Analysis of histone deacetylase (HDAC) mRNA expression levels in the PBMCs of 48 RA patients revealed a significant reduction and negative association with disease characteristics. Therefore, HDAC mRNA might play an essential role in the pathogenesis of RA (68). The single immunoglobulin IL-1-related receptor (SIGIRR) mRNA expression was decreased in the PBMCs of RA patients in a study including 79 such patients, and SIGIRR dysregulation might be related to RA pathogenesis and susceptibility (69). In a recently published study of 650 patients with RA, signaling lymphocyte activation molecule family 6 (SLAMF6) expression in the synovial tissue was 1.6-fold higher than in the controls and correlated with the severity and susceptibility of RA (70). An analysis showed that mRNA expression of the inflammasome genes NOD-like receptor family pyrin domain containing 3 (*NLRP3*) and caspase recruitment domain-containing protein 8 (*CARD8*) in the PBMCs of 230 RA patients from two different populations was correlated with susceptibility and RA progression (*p* = 0.044) and with severity (*p* = 0.03), respectively; in addition, the NLRP3 expression levels were also significantly elevated (71).

One study showed that serum mRNA expression levels of YT521-B homology domains 2 (YTHDF2), alkylation repair homolog protein 5 (ALKBH5), and FTO from a population of 79 RA patients were significantly decreased (*p* < 0.05). The expression of ALKBH5 mRNA was significantly upregulated after regular treatment (therapeutic regimens with corticosteroids and immunosuppressive drugs) (72). FTO mRNA expression occurs in association with DAS28, IgG, complement 3 (C3), and lymphocyte-to-monocyte ratio (LMR), and YTHDF2 mRNA expression was correlated with red blood cell count (RBC), neutrophil-to-lymphocyte ratio (NLR), LMR, lymphocyte percentage (L%), and neutrophil counts (N%) (72). The serum mRNA levels of ribonucleotide reductase subunit M2 (RRM2) were elevated, and the PBMCs of RA patients had an area under the curve (AUC) of 0.941 (*p* < 0.0001; sensitivity = 86.7%; specificity = 90.4%); in addition, significant correlations were observed between RRM2 and DAS-28, CDAI, and swollen and tender joints (73). Furthermore, a study comprising two cohorts with 17 RA patients showed that transforming growth factor beta receptors II (TGFBR2) was lacking in PBMCs, and the expression level of TGFBR2 mRNA might reflect RA disease activity (74). An analysis of 38 female RA patients revealed that CD40 ligand (CD40L) mRNA was overexpressed (*p* < 0.0001) and showed a clear correlation with clinical activity when the data were stratified per DAS28 and a progressive increase in CD40L expression (75). Another study analyzed plasma IL-38 mRNA expression levels in RA patients in a training cohort that included 130 RA patients and a validation cohort of 250 RA patients, respectively, showing that the levels were significantly higher in the RA patient group. In addition, its expression levels correlated with inflammatory parameters at baseline and in subsequent studies, and treatment significantly decreased IL-38 expression, suggesting that IL-38 might be a potential biomarker for RA (sensitivity = 0.723, specificity = 0.906, and AUC = 0.840) (76).

CD26 mRNA expression was found to be 1.68 times higher in RA patients compared with controls (*p* = 0.001), and there was a strong positive association between DAS28 (*p* = 0.002) and bone erosion in the hands (*p* = 0.049) (77). In a study including 104 RA patients, FURIN mRNA expression was significantly increased in the peripheral blood of RA patients (*p* < 0.001), and this was positively correlated with TGF-β1, RF, and anti-CCP (78). Another study comprising 187 patients with RA showed that serum IL-10 mRNA expression was 3.63-fold higher than in controls. There appeared to be a significantly positive correlation with anti-CCP, RF, and CRP (79).

One study comprising 74 RA patients found that YTHDF2 mRNA expression was significantly decreased in RA PBMCs and negatively associated with IL-1β, CRP, ESR, white blood cell counts (WBC), neutrophil counts (N), N%, and NLR values but was correlated with RF and the treatment response (80). G−protein−coupled bile acid receptor 1 (TGR5) mRNA expression was significantly decreased (*p* < 0.001) in RA PBMCs (*n* = 50), and there was a negative correlation between DAS28 (*p* = 0.006) and CRP (*p* = 0.002) (81). Furthermore, IL-35 mRNA expression and Treg frequency were significantly lower in RA patients (*n* = 55) than HCs (*n* = 20), and IL-35 levels were negatively associated with ESR and DAS28, suggesting that IL-35 and Tregs play a protective role in the development of RA (82).

Microarray analysis revealed that class 3 and 4 semaphorins and their receptors are overexpressed in RA patients. The serum mRNA levels of semaphorins were associated with the levels of proangiogenic and inflammatory markers, thus identifying them as therapeutic candidates and potential biomarkers for RA (83). The PBMC levels of laminin receptor 1 (LAMR1) mRNA are downregulated in early RA patients and might be an independent predictor of poor anti-TNF-α therapy response; in addition, these levels are associated with increased disease activity scores (84). IL-32 mRNA expression was higher in PBMCs from RA patients compared with healthy individuals and might play a role in predicting the response to anti-TNF-α therapy (85). FPGS 8PR/8WT ratios in the whole blood of RA patients might have a predictive value for the treatment response to MTX, with higher baseline ratios tending toward a poorer treatment response and higher DAS44 scores (86). Similarly, the whole blood mRNA levels of adenosine A3 receptor (ADORA3) in RA patients were correlated with a non-response to MTX therapy (AUC = 0.7, *p* = 0.006), and the baseline expression levels of ADORA3 mRNA might be a predictive biomarker of MTX response (87).

In summary, the abnormal expression of mRNAs in peripheral blood, plasma, serum, PBMCs, synovial tissue, and T cells of RA patients has potential application prospects for the early diagnosis, prognostic assessment, disease activity, and treatment response monitoring in RA. These examples demonstrate that mRNA expression patterns are, to some extent, potentially disease-specific but still have limitations. To date, the number of conducted studies remains small, and the lack of high-quality studies inevitably reduces their credibility. Moreover, the biofluids and tissues involved in these studies remain limited. Future studies should include urine, meniscus, and macrophages, among other factors. These potential mRNA-based biomarkers are summarized in **Table 1**.

**Table 1:** Potential mRNA biomarkers for RA.

mRNA	Source	Profiling technique	Expression	Application/potential mechanism	RA/HCs	Ref
SLAMF6	Synovial tissue	RT-qPCR	↑	Associated with both the susceptibility and severity of RA	50/40	(70)
MAGE-1	Synovial fluid cell	RT-qPCR, ELISA	↑	Utilized as a diagnostic biomarker and improved the early diagnostic ability of RA combined with RF, anti-CCP	135/78	(88)
CD40L	CD4^+^ T cell	RT-qPCR, ELISA, flow cytometry	↑	May serve as a marker of clinical activity	38/10	(75)
FPGS	Whole blood	RT-qPCR	↑	Ratios of 8PR/8WT as a predictive biomarker for MTX response	36/–	(86)
ADORA3	Whole blood	RT-qPCR	↓	May serve as a biomarker of response to MTX	140/–	(87)
ALKBH5, FTO, YTHDF2	Peripheral blood	RT-qPCR	↓	Associated with autoantibody production and disease activity and may be a promising biomarker	79/61	(72)
IL-38	Plasma	RT-qPCR	↑	Correlated with RA disease activity and may be a promising diagnostic biomarker	250/60	(76)
HLA-DP	Serum	PCR-HRM	Detectable	Correlated to increased risk of RA and elevated serum anti-CCP level	254/391	(89)
IL-10	Serum	RT-qPCR, ELISA	↑	Associated with genetic susceptibility/predisposition to RA	187/214	(79)
NLRP3, CARD8	PBMC	RT-qPCR	↑, detectable	Associated to RA susceptibility and severity	218/307, 12/10	(71, 90)
TGR5	PBMC	RT-qPCR	↓	Negatively correlated with the levels of CRP and DAS28. Attenuates the expression of TNF-α, IL-1β, IL-6, and IL-8 *via* inhibition of NF-κB activity	50/40	(81)
HDAC	PBMC	RT-qPCR, WB	↓	Activity levels and histone H3 acetylation status as a potential biomarker of disease activity	48/48	(68)
YTHDF2	PBMC	RT-qPCR	↓	May have a regulatory role in the underlying mechanisms in RA. Regulates mRNA degradation and translation as m6A reader	74/63	(80)
SOCS1	PBMC	RT-qPCR	↓	Associated with disease progression, disease severity, and response to treatment	138/–	(66)
ABCG2	PBMC	RT-qPCR	↑	Decreased expression is associated with good response to MTX in RA patients	24/–	(91)
IL-32	PBMC	RT-qPCR, ELISA	↑	Play a role in predicting response to RA anti-TNF-α therapy	22/7	(85)
TTP	PBMC	RT-qPCR	↓	Dysregulation correlated with the pathogenesis and development of RA and may be a protective factor	36/37	(92)
TGFBR2	PBMC	RT-qPCR	↓	Abundance shows changes linked to RA disease activity	17/9	(74)
CD26	PBMC	RT-qPCR	↑	Associated with disease activity and bone erosion	20/40	(77)
SIGIRR	PBMC	RT-qPCR	↑	Dysregulation might be associated with the pathogenesis and susceptibility of RA	79/76	(69)
HK2	Serum/PBMC	RT-qPCR	↑	Biomarker for diagnosing RA and involved in disease activity in RA	65/40	(67)
RRM2	Serum/PBMC	RT-qPCR	↑	Showed high diagnosis efficiency for RA patients and is a candidate biomarker	47/40	(73)
FURIN	Serum/PBMC	RT-qPCR, ELISA, WB	↑	Positively correlated with TGF-β1, RF, anti-CCP	108/39	(78)
IL-35	Serum/PBMC	RT-qPCR, ELISA	↓	Negatively correlated with the ESR and DAS28 of RA patients	55/20	(82)
Sema3A	Serum/PBMC	RT-qPCR, ELISA	↑	Correlated with autoantibody production and bone destruction positively and with ESR, IgM, and RF	130/150	(63)
LAMR1	Synovial tissue/PBMC	RT-qPCR, flow cytometry, IH	↓	Lower expression correlates with increased RA activity scores and the pathogenesis of RA. Regulates the threshold and amplitude of cytokine activation and migration	22/25, 20/10	(84)
RPN2	Plasma/PBMC/T cell	RT-qPCR, ELISA	↑	May serve as a novel diagnostic biomarker. Influences the growth and activation of T lymphocytes	35/35	(64)
IL-37	Plasma/PBMC/FLS	RT-qPCR, ELISA	↑	Correlated with disease activity and may be a diagnostic biomarker	230/60	(65, 93)
SEMA	Serum/synovial tissue/endothelial cell	RT-qPCR, ELISA, WB, IH, IF	↑	Identified class 3 and class 4 semaphorins as potential biomarkers and therapeutic candidates in RA	200/30	(83)

↑, upregulated; ↓, downregulated; –, not available; FLS, fibroblast-like synoviocytes; PBMC, peripheral blood mononuclear cell; RT-qPCR, reverse transcription quantitative PCR; WB, Western blot; ELISA, enzyme-linked immunosorbent assay; IH, immunohistochemistry; IF, immunofluorescence.

### miRNAs as biomarkers for RA

miRNAs are small endogenous ncRNAs of 18–24 nucleotides in length that participate in the post-transcriptional regulation of gene expression (94). miRNAs can act as inhibitory regulators by inhibiting the translation or degradation of mRNAs and can also increase the expression of target genes by improving the translation rates (95). They are essential for developing the immune system and regulation (96). In addition, miRNAs have high tissue specificity and are expressed differentially in various tissues (97). An analysis of circulatory miRNAs comprising 50 RA patients showed that miR-126-3p, miR-221-3p, let-7d-5p, miR-431-3p, miR-24-3p, and miR-130a-3p were significantly elevated in the serum of RA patients and “at-risk individuals,” as well as miR-130a-3p combined with the remaining five to yield a higher AUC. Both let-7i-5p and miR-339-5p are significantly decreased post-MTX, which may help in the early diagnosis of RA and monitoring of treatment response or risk of recurrence (98). miR-223 serum expression levels are significantly upregulated in RA patients and could distinguish RA patients from HCs with the AUC (0.85), serving as a potential biomarker for RA diagnosis and risk prediction (99).

An analysis using a next-generation sequencing approach suggested that serum levels of miR-16-5p and miR-223-3p were significantly lower in early RA patients than in established RA patients and HCs and were involved in the pathophysiology of RA. Furthermore, miR-16-5p and miR-223-3p could serve as biomarkers and possible predictors of disease outcomes for early RA (100). The expression of miR-224, miR-483-5p, miR-760, miR-375, and miR-378 is significantly upregulated in the serum of RA patients (*n* = 80) compared with controls (*p* < 0.05), and there is a significantly positive association between these miRNAs and DAS28 scores (*p* < 0.001), suggesting that the serum expression of these miRNAs could be used as biomarkers for the early diagnosis of RA and targets for therapy (101).

The expression of miR-146a was found to be significantly elevated in the peripheral blood of RA patients (*n* = 76) and positively correlated with RA severity, retinoic acid-related orphan receptor variant 2 (RORc), IL-17 levels, and the Th17 cell ratio, yet significantly negatively associated with the Treg cell ratio, TGF-β1, and forkhead box protein 3 (FOXP3) levels, suggesting that it may serve as a biomarker for disease progression and prognosis in RA patients (102). miR-361-5p is significantly more highly expressed in whole blood from early RA patients, with ROC analysis showing AUC = 0.76 and *p* < 0.05, identifying it as a potential biomarker for early RA (103).

A study including 125 patients with RA showed that circulating plasma miR-155 levels were significantly downregulated in RA patients compared with HCs. In contrast, the levels of whole blood miR-155 gene methylation were upregulated, suggesting that these were potentially helpful biomarkers for RA diagnosis (104). miR-146a-5p, miR-125a-5p, and miR-24-3p were upregulated in the plasma of RA patients, and their expression was significantly different in the subgroups of RA patients with varying disease activity. ROC curve analysis indicated good AUC values, sensitivity, and specificity for all three miRNAs, suggesting that these miRNAs could be used as biomarkers for RA diagnosis and disease activity (105). The expression of miR-22-3p and let-7a-5p was significantly upregulated in the plasma of RA patients, which could identify the RA populations and, in combination with anti-CCP and RF, could improve the diagnostic ability of RA (especially seronegative RA) (106).

miR-23b levels were found to be significantly elevated in the synovial tissue cells and plasma of RA patients and positively correlated with platelet (PLT) counts, CRP, hypersensitive-CRP (hs-CRP), ESR, and DAS28 (*p* < 0.05), and treatment reversed the trend of elevated plasma miR-23b levels (107). Moreover, miR-23 could regulate CXCL12 through the NF-κB signaling pathway to suppress the inflammation involved in RA pathogenesis (108). The miR-5571-3p and miR-135b-5p levels in the synovial tissues of RA patients were positively associated with disease activity and the inflammation level, with an AUC of 0.833 when the two were combined and had a good predictive value for RA risk (109).

An analysis of 79 RA patients revealed that serum exosome-encapsulated miR-6089 was significantly reduced in RA patients and may regulate inflammatory responses by directly targeting TLR4 signaling (110). miR-204-5p expression was downregulated in the plasma exosomes of RA patients. It was inversely associated with disease parameters (e.g., RF, CRP, and ESR), which translates communication between immune cells and FLSs and could be used as a potential biomarker for the diagnosis and treatment of RA (111). miR-451a and miR-25-3p are significantly elevated in serum exosomes (secretory extracellular vesicles) from patients with early RA, and when combined with soluble tumor necrosis factor-like weak inducer of apoptosis (sTWEAK), they correctly distinguish 95.6% of patients (ROC = 0.983, specificity = 100%, and sensitivity = 85.7%); they could be used as a panel of serum biomarkers for early RA diagnosis (112). The levels of miR-548a-3p in serum exosomes and PBMCs of RA patients are significantly downregulated and negatively correlated with the levels of RF, ESR, and CRP, suggesting that the miR-548a-3p/TLR4/NF-κB axis could be used as a biomarker for RA diagnosis and targets for therapy (113). Serum exosome miR-1915-3p expression is significantly elevated in RA patients with clinical remission and negatively associated with CRP levels, which may be a potential biomarker of disease activity in Korean RA patients (114). Exosomes participate in cell-to-cell communication *via* the packaging and shuttling of diverse cargo molecules (including miRNAs) to recipient cells and have a crucial role in autoimmune-related disorders (115, 116). In addition, miRNA cargo of exosomes has shown potential diagnostic value as biomarkers in several autoimmune diseases (117).

Notably, hsa-miR-146a-5p, hsa-miR-132-3p, and hsa-miR-155-5p were found to be expressed at high levels in the whole blood of RA patients (*n* = 94). Baseline levels of all three miRNAs were reduced in responders compared with non-responders post-MTX. They were also shown to be potential biomarkers of response to MTX treatment by ROC curve analysis (118). miR-29, miR-26b, miR-522, and miR-451 are significantly differentially expressed in responders compared with non-responders to olokizumab treatment in the plasma of RA patients. ROC curve and regression analyses showed that all four miRNAs were statistically associated with olokizumab treatment efficiency scores and might be potential biomarkers of therapeutic response (119). One study showed no direct effect of tofacitinib treatment on measured miRNA expression in RA patients but found that changes in has-miR-194-5p and has-miR-432-5p might be correlated with proinflammatory pathway regulation and RA flare-ups (120). Another study of 96 RA patients showed a significant upregulation of miRNA-125a and miRNA-125b expression in the plasma of RA patients, which was positively correlated with CRP and tender joint count (TJC), swollen joint count (SJC), ESR, CRR, and DAS28-ESR. The biomarker expression was gradually decreased post-infliximab and was significantly higher in responders at baseline, suggesting that these biomarkers indicate disease activity and response to infliximab treatment (121).

In conclusion, the aberrant expression of miRNAs in the peripheral blood, plasma, serum, PBMCs, synovial tissue, and exosomes of RA patients provides promising new directions for early diagnosis, prognostic assessment, disease activity, and treatment response monitoring in RA. Studies of ncRNAs in RA have focused on miRNAs and have concentrated on circulating biofluids, available through minimally invasive blood draws. These examples suggest that miRNA expression patterns are, to some extent, not only body fluid- or tissue-specific but may also be disease-specific. However, the selection of participants should consider the use of appropriate inclusion and exclusion criteria to facilitate the interpretation of study results and to combine them with other studies for more in-depth analysis. The potential miRNA-based biomarkers for the diagnostic and prognostic assessment of RA are summarized in **Table 2**.

**Table 2:** Potential miRNA biomarkers for RA.

miRNA	Source	Profiling technique	Expression	Target/signaling	Application/potential mechanism	RA/HCs	Ref
miR-5571-3p, miR-135b-5p	Synovial tissue	RT-qPCR, RNA-seq	↑	Unknown	Correlate with increased RA risk and activity	30/30	(109)
miR-143-3p	Synovial tissue	RT-qPCR	↑	IGF1R, IGFBP5/Ras/p38 MAPK signaling	May be a novel therapeutic target in RA. Regulates cell proliferation and apoptosis by targeting IGF1R and IGFBP5 expression and regulating the Ras/p38 MAPK signaling pathways	5/1	(122)
miR-192	Synovial tissue/FLS	RT-qPCR, Luciferase reporter assay, WB	↓	CAV1	The miR-192/CAV1 pathway may represent a novel target for the prevention and treatment of RA	22/10	(123)
miR-23b	Synovial tissue/FLS/plasma	RT-qPCR, microarray, *in-situ* hybridization	↑	Unknown	May be a promising biomarker for the degree of inflammatory disease activities and therapeutic effects in RA	8/4	(107)
miR-23	Synovial tissue/serum	RT-qPCR	↓	CXCL12, NF-κB signaling	Potential target for the diagnosis and treatment of RA. Inhibits inflammation by regulating CXCL12 *via* the NF-κB signaling pathway	22/22	(108)
miR-539	Joint fluid/peripheral blood	RT-qPCR	↓	OPN	Potential biomarker in minimally invasive diagnoses of RA. Promotes the development and progression of RA by regulating osteopontin	68/46	(124)
miR-125a-5p	Peripheral blood	RT-qPCR	↑	Unknown	May serve as a therapeutic response biomarker and used as a target for therapeutic interventions	90/30	(125)
miRNA-146a	Peripheral blood	RT-qPCR	↑	Unknown	May serve as a biomarker for disease progression and prognosis in RA	76/40	(102)
miR-361-5p	Whole blood	RT-qPCR, microarray	↑	Unknown	Could be important for RA pathogenesis and as a biomarker for early RA	20/–	(103)
miR-146a-5p, miR-132-3p, miR-155-5p	Whole blood	RT-qPCR	↑	Unknown	Potential biomarkers of responsiveness to MTX therapy	91/–	(118)
miR-155	Whole blood/plasma	RT-qPCR	↓	Unknown	Host gene methylation status or plasma level might be a potentially useful marker in RA	135/30	(104)
miRNA-146a	Serum	RT-qPCR	↑	Unknown	As a potential prognostic biomarker, may have a role as a therapeutic target	40/40	(126)
miRNA-146a, miRNA-499	Serum	RT-qPCR	↑	Unknown	Used as diagnostic markers for RA patients	52/56	(127)
miRNA-5196	Serum	RT-qPCR	↑	Unknown	Promising as a good biomarker to predict and monitor anti-TNF-α response	10/12	(128)
miR-223	Serum	RT-qPCR, ELISA	↑	Unknown	Could serve as potential biomarkers of RA and as a predictor of RA risk	120/130	(99)
miR-326, miR-495	Serum	RT-qPCR	↓, ↑	Unknown	Combined detection of the two has good diagnostic value for RA	107/112	(129)
miR-155, miR-210	Serum	RT-qPCR	↑, ↓	NF-κB signaling	May serve as independent and non-invasive biomarkers for the diagnosis and disease activity of RA	100/100	(130)
miR-223-3p, miR-16-5p	Serum	Next-generation sequencing	↓	Unknown	Could be used as biomarkers and possible predictors of disease outcome for early RA	54/36	(100)
miR-10a	Serum	RT-qPCR	↓	Unknown	May serve as a biomarker of RA diagnosis and predictor of therapy effectiveness (MTX)	30/30	(131)
hsa-miR-432-5p, hsa-miR-194-5p	Serum	RT-PCR, flow cytometry	↑	SOCS5/NF-κB signaling, –	Associated with the regulation of proinflammatory pathways and RA flare-up	16/–	(120)
miR-224, miR-483-5p, miR-760, miR-375, miR-378	Serum	RT-qPCR	↑	Unknown	Achieved early detection of RA and may be used as targets for treatment	80/80	(101)
miR-126-3p, let-7d-5p, miR-431-3p, miR-221-3p, miR-24-3p, miR-130a-3p	Serum	Flow cytometry-fluorescent	↑	STAT-1, STAT-3, IRF-1, NF-κB, BCL-6	May have potential predictive value for disease onset and early progression	50/20	(98)
miR-22-3p, let-7a-5p	Plasma	RT-qPCR	↑	Unknown	Potential promising diagnostic biomarkers for RA	76/36	(106)
miR-22	Plasma	RT-qPCR	↑	Unknown	May be considered as a potential molecular marker associated with disease activity	50/24	(132)
miR-27a-3p	Plasma	RT-qPCR	↑	Unknown	Potential predictive biomarker of ACR/EULAR remission in patients with early RA (adalimumab & MTX)	180/–	(133)
miR-146a-5p, miR-125a-5p, miR-24-3p	Plasma	RT-qPCR	↑	Unknown	Could be used as suitable biomarkers for RA diagnosis	50/50	(105)
miR-29, miR-26b, miR-522, miR-451	Plasma	RT-qPCR	↑	IL-6/IL-6R signaling	May be a potential therapeutic response biomarker (olokizumab)	103/–	(119)
miRNA-125a, miRNA-125b	Plasma	RT-qPCR	↑	NF-κB signaling	Display the potency for guiding personalized treatment strategy and improving clinical outcomes in RA patients	96/96	(121)
miR-99b-5p	Plasma	RT-qPCR	↑	Unknown	May serve as a possible predictor for erosion progression in early RA	117/–	(134)
miR-451	Plasma/PBMC	RT-qPCR	↑	CXCL16	Biomarker in the preclinical phase of RA. Regulates CXCL16 expression and affects the inflammatory milieu	36/30	(135)
miR-99b-5p	PBMC	RT-qPCR, microarray	↑	mTOR, RASSF4	Provides novel candidate biomarkers for diagnosis. Targeting and inhibiting the expression of mTOR and RASSF4, inhibiting T-cell apoptosis, stimulating T-cell proliferation, activation, and pro-inflammatory cytokine expression	35/35	(136)
miR-221, miR-222	PBMC	RT-qPCR	↑	Unknown	As new novel non-invasive biomarkers for disease detection	30/20	(137)
miR-103a-3p	PBMC	RT-qPCR	↑	TP53, AGET2	Prognostic biomarker for preclinical RA. Associates with AGO2 within RISC and is known to suppress Dicer	18/12	(138)
miR-146a-5p, let-7a-5p	PBMC	RT-qPCR, microarray	↓, ↑	Unknown	Disclosed a great predictive value for clinical response to TNF inhibitor in RA patients combined with CRP and biologics history	92/–	(139)
miRNA-146b	PBMC	qPCR, next-generation sequencing	↑	RARA	Biomarker predicting pro-inflammatory RA progression and disease activity. Negatively regulates the anti-inflammatory RARA transcript	–	(140)
miR-548a-3p	PBMC/serum exosome	RT-qPCR	↓	TLR4/NF-κB signaling	Promising targets for RA diagnosis and treatment. Upregulates NF-κB mediated inflammation	76/20	(141)
miR-1915-3p	Serum exosome	RT-qPCR, microarray	↓	Unknown	May be a potential marker for Korean RA disease activity	42/–	(114)
miR-451a, miR-25-3p	Serum exosome	RT-qPCR, microarray	↑	YWHAB	May be used in the early clinical diagnosis of RA when combined with sTWEAK	24/24	(112)
miR-6089	Serum exosome	RT-qPCR	↓	TLR4 signaling	May serve as a novel promising biomarker in RA. Regulates the generation of IL-6, IL-29, and TNF-α by targeting and controlling TLR4 signaling	76/20	(110)
miR-520h, miR-548n, miR-498, miR-19b-3p	Serum exosome	RT-qPCR, NanoString profiling technology	↑	Unknown	EV miRNA profiling of RA patients could be used for the detection of diagnostic and predictive biomarkers	3/3	(142)
miR-204-5p	Plasma exosome	RT-qPCR, microarray	↓	ANGPT1, CRKL/ERK/MAPK	Potential biomarker for RA diagnosis and treatment. Mediates the communication between immune cells and synovial fibroblasts	86/90	(111)

↑, upregulated; ↓, downregulated; –, not available; FLS, fibroblast-like synoviocytes; PBMC, peripheral blood mononuclear cell; RT-qPCR, reverse transcription quantitative PCR; RNA-seq, RNA sequencing; WB, Western blot; ELISA, enzyme-linked immunosorbent assay.

### lncRNAs as biomarkers for RA

lncRNA plays a crucial role in different biological processes by interacting with DNA to modulate epigenetic modifications, transcription, post-translational modifications, and protein/RNA stability (143). The ROC curve analysis of the expression of lncRNA TSPEAR-AS2 and its target miR-212-3p in the plasma of 73 RA patients showed that TSPEAR-AS2 expression was significantly downregulated and inversely associated with miR-212-3p levels. Regulation of HFLS apoptosis by the TSPEAR-AS2/miR-212-3p axis is involved in the pathogenesis of RA (144). lnc-ITSN1-2 could be a convincing biomarker for RA diagnosis and monitoring of disease activity as it is significantly upregulated in the plasma and synovial tissues of RA patients and positively correlated with DAS28, ESR, and CRP. Notably, the ROC curve analysis showed that lnc-ITSN1-2 had good diagnostic value (AUC = 0.898, specificity = 80%, and sensitivity = 90%) (145, 146).

The expression levels of HOX transcript antisense intergenic RNA (HOTAIR) and lnc-Cox2 were found to be significantly higher in the serum of RA patients compared with healthy subjects, and the ROC curve indicated that it could distinguish RA patients from other populations, serving as a novel non-invasive biomarker for RA diagnosis (147). LINC00305 expression was significantly upregulated in the serum of RA patients and was positively associated with DAS28, anti-CCP, RF, ESR, and CRP. In addition, patients carrying the LINC00305 AT and TT genotypes (rs2850711 polymorphism) had significantly increased DAS28 scores and LINC00305, NF-κB, and MMP-3 levels, suggesting that LINC00305 and its variant rs2850711 (A/T) might serve as biomarkers for the diagnosis and management of RA (148). PlncRNA-1 and its target TGF-β1 expression are significantly decreased and positively correlated in the serum and FLSs of patients with active RA compared with HCs. The levels of plncRNA-1 could differentiate active RA patients from other populations, and it may be involved in the pathogenesis of RA by regulating TGF-β1 (149). Based on the results of ROC analysis, OSER1-AS1 levels in serum and synovial tissue could differentiate RA from HCs with better specificity and sensitivity than RF and anti-CCP, and OSER1-AS1 could be used as a potentially promising biomarker for diagnosis and treatment (150).

RNA sequencing and qPCR validation analysis showed that lnc-AL928768.3 and lnc-AC091493.1 expression levels were elevated in the synovial tissues of RA patients and positively correlated with DAS28-ESR and CRP, which when combined with ROC curve analysis suggested that they are good biomarkers for predicting RA risk and disease activity (151). LINK-A was significantly highly expressed in synovial tissues and FLSs of RA patients and positively associated with the severity of synovitis in RA patients. LINK-A regulates RA FLS invasion and inflammation through HIF-1α and/or miR-1262 pathways, which might be a promising therapeutic target for RA (152). lncRNA growth arrest-specific transcript 5 (GAS5) is significantly downregulated in synovial tissues, serum, and PBMCs of RA patients compared with HCs and negatively correlated with IL6, IL-17, CRP, ESR, DAS28, and anti-CCP, suggesting that it could be used as a potential biomarker for RA diagnosis (153–156). Interestingly, lncRNAs GAS5 (3.31-fold), RNA component of mitochondrial RNA-processing endoribonuclease (RMRP) (2.43-fold), and TNF-α and heterogeneous nuclear ribonucleoprotein L (THRIL) (2.14-fold) were significantly upregulated in the circulating T cells of RA patients compared with controls, and the ROC curve analysis of the three indicated their value in discriminating RA patients from controls (157).

The expression level of RP11-83J16.1 was found to be increased in the synovial fluid of RA patients, which correlated with increased disease activity and inflammation in RA patients (158). Maternally expressed gene 3 (MEG3) expression was downregulated, and metastasis-associated lung adenocarcinoma transcript 1 (MALAT1) and nuclear enriched abundant transcript 1 (NEAT1) expression were upregulated in the synovial fluid, plasma, and PBMCs of RA patients, and MEG3 and NEAT1 with TJC, NEAT1 with SJC, and DAS28-CRP showed significant correlations, suggesting that they might be used as biomarkers to monitor disease activity (159). Another study showed that MEG3 in PBMCs was negatively associated with disease activity, lesion joints, and inflammation in RA patients (*n* = 191), which could be used as a biomarker in monitoring the treatment efficacy of RA (160).

Compared with HCs, the expression of ENST00000619282 and MIR22HG was found to be upregulated in the PBMCs of RA patients. However, the expression of DSCR9, MAPKAPK5-AS1, and LINC01189 was downregulated. These five lncRNAs were associated with patients’ self-perception and with their clinical indexes (e.g., RF, IgA, IgG, and C3). The ROC curve analysis suggested that these lncRNAs were correlated with apoptosis and autophagy and could be used as promising biomarkers for diagnosing and monitoring RA progression (161). LINC00638 levels were significantly reduced in the PBMCs of RA patients (*n* = 45) compared with normal controls. The levels were negatively associated with DAS28, ROS, IL-17, and ESR, which might inhibit inflammation and oxidative stress by activating the Nrf2/HO-1 pathway (162).

The upregulation of lnc-NEAT1 levels in the PBMCs of RA patients was found to be negatively associated with the expression levels of its targets (miR-125a and miR-21). They were significantly associated with ESR, CRP, and DAS28-ESR scores, and lnc-NEAT1 expression levels were significantly decreased in remission compared with non-remission patients; these biomarkers might indicate RA treatment efficacy and disease activity (163). The lnc-RNU12 expression levels were significantly downregulated in the PBMCs and T-cell subsets of RA patients. This finding suggested that these biomarkers might be involved in the pathogenesis of RA by targeting cyclin L2 (CCNL2) and c-JUN, which affect the T-cell cycle (164). The expression levels of LINC00304, LINC01504, FAM95B1, and lncRNAs were decreased in the PBMCs of RA patients, but the MIR503HG level was increased. Based on the correlation analysis, these lncRNAs were correlated with clinical or laboratory indicators such as disease duration, joint tenderness, arthrocele, RF, and IgG. The lncRNAs might be potential biomarkers for diagnosing RA (165).

The clinical response prediction model comprising lncRNAs RP3-466P17.2, RP11-20D14.6, RP11-844P9.2, and TAS2R64P in PBMCs showed good predictive capability for the etanercept treatment response (AUC = 0.956). This finding suggests that they might be useful biomarkers for the response to etanercept treatment in RA patients (166).

Therefore, the abnormal expression of lncRNAs in RA patients’ peripheral blood, plasma, serum, PBMCs, synovial tissue, synovial fluid, and T cells could be promising for early diagnosis, prognostic assessment, disease activity, and treatment response monitoring in RA. Most of these studies above were limited to the differential expression levels of ncRNAs in single biofluids or tissues. However, some suggested that the ncRNAs in the circulation might not be expressed at the same level as in the tissues. Therefore, multilevel analysis is necessary in the future. Currently, there is no consistent profile of ncRNAs identified or validated in RA studies, and the answer is even more unclear for clinical practice. The utility of these ncRNAs as biomarkers requires rigorous large-scale studies. The challenges of this approach include how to define patient groups, disease characteristics across studies, the analytical platforms used, and biofluid handling measures, which are unresolved and make it difficult to conduct direct comparisons of the findings across studies. These potential lncRNA-based biomarkers for RA diagnosis and prognosis are summarized in **Table 3**.

**Table 3:** Potential lncRNA biomarkers for RA.

lncRNA	Source	Profiling technique	Expression	Target/signaling	Application/potential mechanism	RA/HCs	Ref
lnc-AL928768.3, lnc-AC091493.1	Synovial tissue	RT-qPCR, RNA-seq	↑	Unknown	Novel biomarkers for RA risk and activity	30/30	(151)
GAS5	Synovial tissue	RT-qPCR	↓	miR-128-3p/HDAC4 axis	Suggesting a potential lncRNA-targeted therapy for RA treatment. Regulates HDAC4 *via* miR-128-3p to restrain inflammation in synovial tissue	40/20	(154)
lnc-ITSN1-2	Synovial tissue	RT-qPCR, RNA-seq	↑	NOD2/RIP2 signaling	Positively correlated with disease risk, inflammation, and activity of RA. Regulates the NOD2/RIP2 signaling pathway to reduce RA FLS proliferation and inflammation	30/15	(146)
GAPLINC	FLS	RT-qPCR	↑	miR-382-5p, miR-575	May provide a novel valuable therapeutic target for RA. Promotes a tumor-like behavior of RA-FLS in an miR-382-5p- and miR-575-dependent manner	11/3	(167)
RP11-83J16.1	Synovial fluid/FLS	RT-qPCR, RNA-seq	↑	URI1, β-catenin signaling	Correlates with increased risk and disease activity of RA	25/25	(168)
PICSAR	Synovial fluid/FLS	RT-qPCR, microarray	↑	miR-4701-5p	May act as a biomarker of RA. Promotes synovial invasion and joint destruction by sponging miR-4701-5p	14/8	(169)
MEG3, MALAT1, NEAT1	Synovial fluid/plasma/PBMC	RT-qPCR	↓, ↑, ↑	Unknown	May be probable markers in monitoring disease activity	106/25, 191/-	(159, 160)
LINK-A	Synovial tissue/FLS	RT-qPCR, microarray	↑	HIF-1α, miR-1262	May be a potential therapeutic target for RA	30/22	(152)
ZFAS1	Synovial tissue/FLS	RT-qPCR	↑	miR-27a	May be an effective therapeutic target for RA patients	40/40	(170)
lnc-NEAT1	Synovial tissue/PBMC	RT-qPCR	↑	miR-21, miR-125a	May be a potential biomarker to monitor disease activity and treatment outcome in RA	130/60	(163, 171, 172)
OSER1-AS1	Synovial tissue/serum	RT-qPCR	↓	miR-1298-59/E2F1 axis	May be a hopeful diagnostic and therapeutic objective for RA	30/30	(150)
lnc-PVT1	Synovial tissue/serum	RT-qPCR	↑	miR-146a, miR-145-5p	As promising biomarkers for the diagnosis of RA and may have an important role as therapeutic targets for RA	40/40	(173, 174)
FOXD2-AS1	Synovial tissue/serum	RT-qPCR	↑	miR-331-3p/PIAS3 axis	Represents a promising treatment approach. Promotes RA progression by regulating the miR-331-3p/PIAS3 pathway	43/21	(175)
GAS5	Serum	RT-qPCR	↓	Unknown	May serve as a biomarker for the early detection of RA	200/150	(155)
CASC2	Serum	RT-qPCR	↓	miR-18a-5p/BTG3 axis	Could serve as a novel therapeutic option for RA	30/30	(176)
HOTAIR, lnc-Cox2	Serum	RT-qPCR, WB	↑	miR-106b-5p, –	Could be used as novel non-invasive biomarkers for the diagnosis of RA	60/60	(147, 177)
LINC00305	Serum	RT-qPCR	↑	Unknown	May play a role in the diagnosis and management of RA and its severity and activity	100/100	(148)
RNA143598, RNA143596, HIX0032090, IGHCgamma1, XLOC_002730	Serum	RT-qPCR	↑	Unknown	Associated with the disease course, RF, anti-CCP, and ESR of patients with RA	43/40	(178)
GAS5	Serum/FLS	RT-qPCR	↓	miR-222-3p/Sirt1 signaling	As a potential therapeutic strategy for RA progression	35/35	(156)
THRIL	Serum/FLS	RT-qPCR	↑	PI3K/AKT signaling	Playing important roles in promoting the occurrence and development of RA	16/12	(179)
PlncRNA-1	Serum/FLS	RT-qPCR, ELISA	↑	TGF-β1	Serves as a biomarker of active RA patients and participates in RA pathogenesis possibly by regulating TGF-β1	70/40	(149)
TSPEAR-AS2	Plasma	RT-qPCR	↓	miR-212-3p	Showed promising diagnostic value for RA	73/66	(144)
lnc-ITSN1-2	Plasma	RT-qPCR	↑	Unknown	Novel and convincing biomarker for RA diagnosis and disease management	30/30	(145)
DILC	Plasma	RT-qPCR	↓	Unknown	Inversely correlated with IL-6 and may participate in RA by inducing apoptosis of FLS and downregulating IL-6	75/66	(180)
CASC2	Plasma	RT-qPCR	↓	Unknown	Overexpression promotes the apoptosis of HFLSs by downregulating IL-17, thereby suppressing the progression of RA	65/54	(181)
RP11-498C9.15	PBMC	RT-qPCR, microarray	↑	Unknown	May play a pivotal role in RA pathogenesis	20/20	(182)
GAS5	PBMC	RT-qPCR	↓	AMPK signaling	Serves as a potential diagnostic marker for RA. Activates the AMPK pathway	20/20	(153)
E2F3-IT1	PBMC	RT-qPCR	↑	LDLR, PLSCR1, PARP9	May be involved in RA pathogenesis by affecting T‐cell growth and activation	35/35	(183)
RP3-466P17.2, RP11-20D14.6, RP11-844P9.2, TAS2R64P	PBMC	RT-qPCR, RNA-seq	Detectable	Unknown	Potentially a useful tool to instruct etanercept treatment in RA	80/–	(166)
IFNG-AS1	PBMC	RT-qPCR	↑	IFNG	A biomarker combined with RF and anti-CCP could improve the sensitivity and specificity of RA diagnosis	31/30	(184)
MIR503HG, LINC00304, LINC01504, FAM95B1	PBMC	RT-qPCR, RNA-seq	↑, ↓*3	Unknown	May serve as potential biomarkers for RA diagnosis, influencing the occurrence and progress of RA	10/10	(165)
LINC00638	PBMC	RT-qPCR	↓	Nrf2/HO-1 signaling	Associated with immune inflammation, oxidative stress, and disease activity. Inhibit the inflammation and oxidative stress by activating the Nrf2/HO-1 pathway	45/30	(162)
ENST00000619282, MIR22HG, DSCR9, MAPKAPK5-AS1, LINC01189	PBMC	RT-qPCR, RNA-seq	↑*2, ↓*3	Unknown	May serve as potential biomarkers for the diagnosis and monitoring of RA progression	20/20	(161)
lnc-RNU12	PBMC/T-cell subtypes	RT-qPCR, microarray	↓	c-JUN, CCNL2	Involved in the pathogenesis of RA by influencing the T-cell cycle by targeting c-JUN and CCNL2	28/18	(164)
ENST00000420096, ENST00000563752, ENST00000444038, ENST00000572491, ENST00000569543, NR_039985, NR_038238, uc021xin.1, NR_027148	Peripheral blood/CD4^+^ T cell	RT-qPCR, high-throughput sequencing	↑*7, ↓*2	CCL19	Potential value as diagnostic biomarkers for active RA, involved in the pathogenesis of RA and the differentiation of CD4^+^ T cells	12/8	(185)
GAS5, RMRP, THRIL	T cell	RT-qPCR	↑	Unknown	Have a discriminative value in comparing RA patients and other populations	20/18	(157)

↑, upregulated; ↓, downregulated; –, not available; FLS, fibroblast-like synoviocytes; PBMC, peripheral blood mononuclear cell; RT-qPCR, reverse transcription quantitative PCR; RNA-seq, RNA sequencing; WB, Western blot; ELISA, enzyme-linked immunosorbent assay. The meaning of * is the multiplication sign, which represents how many RNAs are up-regulated or downregulated (in order of appearance).

### circRNAs as biomarkers for RA

circRNAs are novel, approximately 500-nt endogenous ncRNAs noted to comprise closed round structures with high stability and are often characterized by tissue-specific expression and evolution-based conservation (186). circRNAs play many roles in various biological processes, including RNA maturation regulation, alternative splicing, protein localization, miRNA sponging, histone modifications, and protein translation (187). The levels of circ_0002715 and circ_0035197 have been found to be significantly elevated in the peripheral blood of RA patients compared with HCs, and circ_0002715 expression correlates with disease duration, RF, ACPA, TJC, and SJC. Studies on ROC curve analysis and logistic regression models have suggested that the combination of circ_0002715 and circ_0035197 might be a biomarker for diagnosis and disease activity in new-onset RA (AUC = 0.758, sensitivity = 72.9%, and specificity = 71.4%). They can differentiate RA patients from patients with SLE or AS and HCs (188).

circ_AFF2 levels were found in one study to be upregulated in the peripheral blood of RA patients, increasing TAB2 expression to promote RA progression by sponging miR-375, which can be used as a biomarker for RA diagnosis and treatment (189). circ-AFF2 overexpression induced an inflammatory response, proliferation, migration, and invasion of RA FLSs through regulation of the miR-650/CNP axis (190). A study including 77 RA patients showed that circ_0044235 was significantly downregulated in the peripheral blood of RA patients and might specifically identify RA patients from SLE patients. This finding suggests that circ_0044235 could serve as a potential biomarker for diagnosing RA patients (AUC = 0.779) (191). In addition, circ_0044235 is involved in RA development by promoting SIRT1 expression through sponge miR-135b-5p, which acts on the NLRP3-mediated pyroptosis pathway (192). circ_0005198 and circ_0005008 have been found to be significantly upregulated in the plasma from new-onset RA patients compared with SLE patients and HCs when evaluated by microarray and RT-qPCR analysis. These biomarkers are positively correlated with DAS28, RF, CRP, and ESR levels, suggesting that the circRNAs can be used as biomarkers of diagnosis (AUC = 0.783; 0.829) and disease activity for new-onset RA (193). The expression of circHIPK3 was found to be significantly upregulated in the serum of RA patients. It might be involved in RA pathogenesis by increasing monocyte chemotactic protein-1 (MCP-1) secretion through interactions with miRNA-124a to induce joint inflammation (194).

According to the ROC curve analysis, the diagnostic value of circPTPN22 could discriminate RA patients from SLE patients and HCs (AUC = 0.781; 0.934). circPTPN22 levels were found to be significantly downregulated in the PBMCs of RA patients and negatively correlated with RF, anti-CCP, CRP, IgA, IgM, and IgG levels. Further analysis suggested that this may be a potential biomarker for the diagnosis of RA and is involved in RA’s pathogenesis (195). ciRS-7 expression was significantly elevated in the PBMCs of RA patients and may potentially distinguish RA patients from HCs (AUC = 0.766). In addition, ciRS-7 sponges may relieve the inhibitory effect on mTOR by adsorbing miR-7 (196). The expression of hsa_circ_0140271 was found to be significantly upregulated in the PBMCs of female RA patients and positively associated with antistreptolysin (ASO). The hsa_circ_0140271 could discriminate female RA patients from those from populations with AS or OA and HCs, according to ROC curve analysis, which could increase diagnostic accuracy when combined with anti-CCP (AUC = 0.818) (197).

The expression levels of circNUP214 in PBMCs could distinguish RA patients from HCs (AUC = 0.76, sensitivity = 42.86%, and specificity = 96.43%). circNUP214 is highly expressed in RA patients and is positively correlated with serum anti-CCP and IL-23 receptor (IL-23R) expression levels. It is also involved in RA pathogenesis by regulating IL-23R in RA patients to promote Th17 cell response (198). The expression levels of circ_0000396 and circ_0130438 in the PBMCs of RA patients (*n* = 36) could serve as potential biomarkers for RA diagnosis, and they are significantly reduced in RA patients compared with HCs (199). Notably, circ_0008410 was significantly upregulated in the PBMCs of RA patients, while circ_0000175 was downregulated, and their expression levels were correlated with RA disease activity and severity. ROC curve analysis showed that the combination of both can improve the accuracy of RA diagnosis (AUC = 0.971, sensitivity = 93.10%, and specificity = 93.33%) and can distinguish RA patients from AS and SLE patients (200). hsa_circ_101328 was found to be significantly decreased in the PBMCs of RA patients and inversely associated with CRP. The ROC curve analysis (AUC = 0.957, sensitivity = 95.2%, and specificity = 95%) indicated that it might be an effective biomarker for RA diagnosis (201). In addition, circ_0001200, circ_0001566, circ_0003972, and circ_0008360 were significantly differentially expressed in the PBMCs of RA patients. These circRNAs were also significantly associated with clinical indicators of patient disease severity (e.g., DAS28, joint induration, and anti-CCP, IgG), which could serve as biomarkers for RA diagnosis (202).

Therefore, the abnormal expression of circRNAs in RA patients’ peripheral blood, plasma, serum, and PBMCs may be significant in early diagnosis, prognostic assessment, disease activity, and treatment response monitoring in RA. Circulating levels of lncRNAs or circRNAs can function as sponges of miRNA and protein or scaffolds for translation. lncRNAs and circRNAs can act by sponging miRNAs and consequently blocking their activity, and this sponging is also the mechanism by which different types of ncRNAs can interact. circRNAs can function by sponging miRNAs to reduce the number of miRNAs available to target mRNA, thus contributing to mRNA stability or protein expression. All these mechanisms allow lncRNAs and circRNAs to play an essential role in the differential expression and pathogenesis of RA (57). These potential circRNA-based biomarkers for RA diagnosis and prognosis are summarized in **Table 4**.

**Table 4:** Potential circRNA biomarkers for RA.

circRNA	Source	Profiling technique	Expression	Target/signaling	Application/potential mechanism	RA/HCs	Ref
circ_0088194	FLS	RT-qPCR, RNA-seq	↑	miR-766-3p/MMP2 axis	Novel therapeutic target to prevent and treat RA. Promotes RA-FLS invasion and migration through the miR-766-3p/MMP2 axis	9/7	(203)
circMAPK9	Synovial tissue/FLS	RT-qPCR	↑	miR-140-3p/PPM1A axis	Novel possible target for RA therapy	22/22	(204)
circRNA_0025908	Synovial tissue/FLS	RT-qPCR	↑	miR-650/SCUBE2 axis	Potential therapeutic clue for RA patients	–	(205)
circ_0003972	Synovial tissue/FLS	RT-qPCR	↑	miR-654-5p/FZD4 axis	Accelerates RA progression by regulating miR-654-5p/FZD4 axis	31/16	(206)
circ_0000396	Synovial tissue/FLS	RT-qPCR	↓	miR-203/HBP1 axis	Potential therapeutic target for RA. Regulates the miR-203/HBP1 axis to inhibit the growth and inflammation in RA FLSs	31/25	(207)
circ_0008360	Synovial tissue/FLS	RT-qPCR	↓	miR-135b-5p/HDAC4 axis	Potential target for the prevention and treatment of RA. Positively regulated HDAC4 expression by sponging miR-135b-5p	–	(208)
circ_AFF2	Synovial tissue/FLS	RT-qPCR	↑	miR-650/CNP axis	May serve as an important intervention for RA therapy. Promotes inflammatory response, proliferation, migration, and invasion of RAFLSs by modulating the miR-650/CNP axis	34/23	(190)
	Peripheral blood			miR-375/TAB2 axis	Biomarker in the diagnosis and treatment of RA. Regulates the expression of TAB2 by targeting miR-375	39/28	(189)
circ_0035197, circ_0002715	Peripheral blood	RT-qPCR	↑	Unknown	Potential biomarker of patients with new-onset RA and associated with disease activity	59/35	(188)
circ_0044235	Peripheral blood/serum	RT-qPCR	↓	miR-135b-5p–SIRT1 axis	Potential biomarker of patients with RA. Acted on the NLRP3-mediated pyroptosis pathway *via* the miR-135b-5p–SIRT1 axis	77/50	(191, 192)
circ_0005198, circ_0005008	Serum	RT-qPCR, microarray hybridization	↑	miR-4778-3p, -	Potential biomarkers of the diagnosis and disease activity for new-onset RA	49/40	(193)
circHIPK3	Serum	RT-qPCR	↑	miRNA-124a	Key players in the pathogenesis of RA. Promote disease severity by inducing MCP-1 secretion *via* sponging miRNA-124a	79/30	(194)
circ_0003353	PBMC	RT-qPCR, RNA-seq	↑	Unknown	Potential biomarkers for the diagnosis of RA	20/20	(209)
ciRS-7	PBMC	RT-qPCR	↑	miR-7/mTOR axis	Suitable biomarker for RA diagnosis. Relieves the inhibitory effect of mTOR by inhibiting miR-7	18/14	(196)
circ_0140271	PBMC	RT-qPCR, RNA-seq	↑	Unknown	Promising diagnostic biomarker for female RA. May regulate fatty acid metabolism pathways in RA by acting as a microRNA sponge	47/47	(197)
circNUP214	PBMC	RT-qPCR	↑	miR-125a-3p, IL-23R	Potential auxiliary indicator of immune disorder in RA. Promotes Th17 cell response by regulating IL-23R	28/28	(198)
circ_0000396, circ_0130438	PBMC	RT-qPCR, RNA-seq	↓	Unknown	Potential diagnosis biomarkers for RA	32/20	(199)
circPTPN22	PBMC	RT-qPCR, high-throughput sequencing	↓	miR-3074-5p, miR-373-3p, miR-766-3p, miR-34c-5p	A novel biomarker for the diagnosis of RA. Involved in RA pathogenesis *via* a sponge mechanism	42/44	(195)
circ_0001200, circ_0001566, circ_0003972, circ_0008360	PBMC	RT-qPCR, RNA-seq	↑*3, ↓	Unknown	Potential biomarkers for the diagnosis of RA	10/10	(202)
circRNA_104871, circRNA_003524, circRNA_101873, circRNA_103047	PBMC	RT-qPCR, microarray hybridization	↑	Unknown	May serve as potential biomarkers for RA diagnosis	30/25	(210)
circ_0008410, circ_0000175	PBMC	RT-qPCR	↑, ↓	Unknown	Potential diagnosis biomarkers for RA and its severity and activity	63/21	(200)
circ_101328	PBMC	RT-qPCR, microarray hybridization	↓	Unknown	Novel and effective biomarker for the early diagnosis of RA	20/20	(201)

↑, up-regulated; ↓, down-regulated; -, not available. FLS: Fibroblast-like synoviocytes, PBMC: Peripheral blood mononuclear cell, RT-qPCR: Reverse Transcription quantitative PCR, RNA-seq: RNA-sequencing. The meaning of * is the multiplication sign, which represents how many RNAs are up-regulated or downregulated (in order of appearance).

### tRNAs, tiRNAs, snoRNAs, piRNAs, and rRNAs as biomarkers for RA

The tRNAs are essential components of the translation machinery that deliver amino acids to the ribosome and synthesize proteins under mRNA guidance. tRNA-encoding genes show tissue-specific and cell-type-specific expression patterns, and dysregulation of tRNAs and tRNA-derived small RNAs (tsRNAs) is involved in pathological processes (211). In addition, tsRNAs are involved in the regulation of rRNA synthesis, mRNA stability, transcription, and RNA reverse transcription and play an important role in cellular functions and in the occurrence and development of various diseases. tsRNAs may be potential biomarkers and therapeutic targets due to their structural stability, high conservation, and extensive distribution (particularly in biofluids, tissues, and exosomes) (212). The snoRNAs are primarily in charge of post-transcriptional modifications, directing the chemical modifications of rRNAs and snRNAs and fine-tuning spliceosome and ribosome function. The dysregulation of snoRNAs, potential biomarkers of disease, in various diseases has been widely reported, and they are potential candidates for biomarkers (213).

piRNAs are probably the most abundant (30,000 members in humans) sncRNAs of 24–31 nt in length, newly identified within the genome, and play important roles in the maintenance of germline integrity, transposon silencing, epigenetic regulation, and post-transcriptional and translational control. Many studies have implicated piRNAs as regulators of various diseases (214). Sequencing analysis of sncRNAs in the sera of DMARD-naive patients receiving 6 months of triple DMARD therapy identified five sncRNAs that were differentially expressed between responders and non-responders at baseline. The baseline expression levels of chr1.tRNA131-GlyCCC, chr2.tRNA13-AlaCGC, chr1.tRNA131-GlyCCC 5′ tiRNA, chr2.tRNA13-AlaCGC 5′ tiRNAs, snoRNA U2-L166, and piR35982 were significantly upregulated in non-responders compared with responders, while rRNA 5S-L612 was the unique sncRNA that was significantly elevated among responders. After treatment, chr1.tRNA131-GlyCCC expression was significantly reduced in ACPA and RF-positive patients and showed a significant positive association with TJC28, suggesting that elevated circulating levels of chr1.tRNA131-GlyCCC 5′ tiRNA may indicate increased inflammation. Similarly, snoRNA U2-L166 was positively correlated with TJC28. In addition, piR-35982 was significantly reduced in RF-positive patients and inversely associated with CRP and ESR levels. These findings suggest that baseline levels of sncRNAs could be a clinically useful biomarker of triple DMARD responsiveness (215). However, there are few studies on applying tRNAs, tsRNAs, snoRNAs, piRNAs, and rRNAs as biomarkers for RA. These potential biomarkers for the diagnostic and prognostic assessment of RA are summarized in **Table 5**.

**Table 5:** Potential tRNA, tiRNA, snoRNA, piRNA, and rRNA biomarkers for RA.

ncRNA	Source	Profiling technique	Expression	Target/signaling	Application/potential mechanism	RA/HCs	Ref
**tRNA:** chr1. tRNA131-GlyCCC, chr2.tRNA13-AlaCGC	Serum	RNA-seq	↑	Unknown	Clinically useful biomarkers of triple DMARD responsiveness	42/–	(215)
**tiRNA:** chr1.tRNA131-GlyCCC 5′ tiRNA, chr2.tRNA13-AlaCGC 5′ tiRNAs							
**snoRNA:** U2-L166							
**piRNA:** piR-35982							
**rRNA:** 5S-L612							

↑, upregulated; –, not available; RNA-seq, RNA sequencing; DMARD, disease-modifying anti-rheumatic drug.

## Conclusion and future outlook

RA is one of the most common and highly heterogeneous autoimmune diseases associated with a considerable increase in disability and mortality (216). Delayed diagnosis is one of the most critical problems in RA management. In the later stages of the disease, patients often experience functional decline and disability or even systemic multi-organ damage (2). Early RA diagnosis and treatment can prevent or significantly delay disease progression in up to 90% of patients, making early diagnosis of RA critical to patient prognosis (4). The 2010 ACR/EULAR criteria have enabled more early RA patients to be diagnosed compared with the 1987 ACR criteria, which are still limited, and many early RA patients are not diagnosed soon enough, thereby missing early disease management (217). Many countries are increasingly focusing on early screening and are exploring and developing less invasive or non-invasive techniques to improve the accuracy of early RA diagnosis. Therefore, significant progress is needed in this area to achieve an early and accurate diagnosis, personalized treatment, and monitoring of RA disease activity and treatment response.

Increasing evidence suggests that ncRNAs play a crucial role in the onset and progression of RA. Studies in transcriptomics and epigenetics and the maturation of high-throughput sequencing technologies have further improved our understanding of RA pathophysiology and pathogenesis. In this review, we described the potential of various RNAs to be promising biomarkers for RA, allowing biofluid biopsies in place of tissue samples and cell line models. Furthermore, not only do individual RNA biomarkers have diagnostic and prognostic value, but also the combined application of multiple RNA biomarkers often exhibits a higher diagnostic and prognostic specificity and sensitivity. The main advantage of RNAs as biomarkers is that they can be detected in various biofluids, which permits a non-invasive diagnosis to be made. There are many studies on RNA biomarkers in RA, but opinion is divided. There is lack of research on tRNA, tsRNA, snoRNA, snRNA, and piRNA as biomarkers for RA. Furthermore, future studies aim to identify which non-invasive diagnostic biomarkers for RA are feasible and cost-effective, to understand which biomarkers can better guide “precision individualized diagnosis and treatment management” of patients, and to better predict patient prognosis.

## Author contributions

YJ and SZ organized the literature and original draft writing. JW and LL contributed to literature retrieval and data collation. SZ, SH, and JW contributed to the manuscript revision. HC and YY were responsible for the conception, writing review, and approval of the submitted version. All authors contributed to the article and approved the submitted version.

## Glossary

**Table:**

RA	rheumatoid arthritis
OA	osteoarthritis
SLE	systemic lupus erythematosus
AS	ankylosing spondylitis
pSS	primary Sjögren’s syndrome
HCs	healthy controls
CRP	C-reactive protein
hs-CRP	hypersensitive C-reactive protein
ESR	erythrocyte sedimentation rate
RF	rheumatoid factor
ACPA	anti-citrullinated protein antibodies
ncRNA	non-coding RNA
anti-CCP	anti-cyclic citrullinated peptides
GWAS	genome-wide association studies
HLA	human leukocyte antigen
SNPs	single nucleotide polymorphisms
MTX	methotrexate
TYMS	thymidylate synthase
DHFR	dihydrofolate reductase
FPGS	folylpolyglutamate synthetase
ENOSF1	enolase superfamily member 1
TNF-α	tumor necrosis factor-α
FTO	fat mass and obesity-associated protein
PTPRC	protein tyrosine phosphatase receptor type C
MMP-3	matrix metalloproteinase-3
sFRβ	soluble folate receptor β
CTX-I	C-terminal telopeptide of collagen type I
sSR-A	soluble scavenger receptor-A
FGL1	fibrinogen-like protein 1
SAA4	serum amyloid A-4 protein
RBP4	retinol-binding protein-4
VDBP	vitamin D-binding protein
AGT	angiotensinogen
PGLYRP-2	peptidoglycan recognition protein-2
LBP	lipopolysaccharide-binding protein
DREAM	dialogue on reverse engineering assessment and methods
BRAF	v-RAF murine sarcoma viral oncogene homolog B
PAD4	peptidylarginine deiminase 4
IL-6	interleukin-6
IL-1β	interleukin-1β
CTGF	connective tissue growth factor
LRG	leucine-rich alpha2 glycoprotein
KL-6	Krebs von den Lungen-6
VCAM1	vascular cell adhesion protein 1
VEGF	vascular endothelial growth factor
CXCL13	C-X-C motif chemokine ligand 13
YKL-40	chitinase-3-like-1 protein
sICAM1	soluble intercellular adhesion molecule-1
IFIH1	interferon-induced helicase gene
IRF5	interferon regulatory factor 5
Sema3A	semaphorin 3A
IgM	immunoglobulin M
RPN2	ribophorin-II
SOCS1	suppressor of cytokine signaling 1
HK2	hexokinase-2
HDAC	histone deacetylase
SIGIRR	single immunoglobulin IL-1-related receptor
SLAMF6	signaling lymphocyte activation molecule family 6
NLRP3	NOD-like receptor family pyrin domain containing 3
CARD8	caspase recruitment domain-containing protein 8
YTHDF2	YT521-B homology domains 2
ALKBH5	alkylation repair homolog protein 5
RRM2	ribonucleotide reductase subunit M2
TGFBR2	transforming growth factor beta receptors II
CD40L	CD40 ligand
TGF-β1	transforming growth factor-β1
TGR5	G−protein−coupled bile acid receptor 1
LAMR1	laminin receptor 1
ADORA3	adenosine A3 receptor
FOXP3	forkhead box protein 3
RORc	retinoic acid-related orphan receptor variant 2
sTWEAK	soluble tumor necrosis factor-like weak inducer of apoptosis
HOTAIR	HOX transcript antisense intergenic RNA
GAS5	growth arrest-specific transcript 5
THRIL	TNF-α and heterogeneous nuclear ribonucleoprotein L
MEG3	maternally expressed gene 3
MALAT1	metastasis-associated lung adenocarcinoma transcript 1
NEAT1	nuclear enriched abundant transcript 1
CCNL2	cyclin L2
MCP-1	monocyte chemotactic protein-1
tRNA	transfer RNA
tsRNA	tRNA-derived small RNA
rRNA	ribosomal RNA
snoRNA	small nucleolar RNA
snRNA	small nuclear RNA
lncRNA	long non-coding RNA
circRNA	circular RNA
miRNA	microRNA
siRNA	small interfering RNA
piRNA	piwi-interacting RNA
mRNA	messenger RNA
PBMCs	peripheral blood mononuclear cells
BMD	bone mineral density
DAS28	28-Joint Disease Activity Score
CDAI	Clinical Disease Activity Index
SDAI	Simplified Disease Activity Index
ROC	receiver operating characteristic
FLSs	fibroblast-like synoviocytes
C3	complement 3
IgA	immunoglobulin A
IgM	immunoglobulin M
IgG	immunoglobulin G
LMR	lymphocyte-to-monocyte ratio
RBC	red blood cell count
NLR	neutrophil-to-lymphocyte ratio
L%	lymphocyte percentage
N%	neutrophil percentage
AUC	area under the curve
WBC	white blood cell counts
N	neutrophil counts
DAS44	44-joint disease activity score
PLT	platelet
28TJC	28-joint tender joint count
28SJC	28-joint swollen joint count
DMARD	disease-modifying anti-rheumatic drug
ASO	antistreptolysin
PCR-HRM	polymerase chain reaction-high-resolution melting
RT-qPCR	reverse transcription quantitative PCR
ELISA	enzyme-linked immunosorbent assay
WB	Western blot
IH	immunohistochemistry
IF	immunofluorescence
RNA-seq	RNA sequencing.
